# Genome-Wide Identification of WRKY Group II Genes and the Role of *HbWRKY11* in *Hordeum brevisubulatum* Under Saline-Alkali Stress

**DOI:** 10.3390/plants15060926

**Published:** 2026-03-17

**Authors:** Sihan Chen, Yicheng Yin, Guangyao Qi, Yanda Li, Bello Hassan Jakada, Dan Sun, Xinying Liu, Xingguo Lan

**Affiliations:** 1 Key Laboratory of Saline-Alkali Vegetation Ecology Restoration, Ministry of Education, College of Life Sciences, Northeast Forestry University, Harbin 150040, China; 2023222087@nefu.edu.cn (S.C.); 2023212071@nefu.edu.cn (Y.Y.); 13406812571@163.com (G.Q.); 2023212037@nefu.edu.cn (Y.L.); bello.jakada@nefu.edu.cn (B.H.J.); 2 Crop Resources Institute, Heilongjiang Academy of Agricultural Sciences, Harbin 150086, China; sundan0906@163.com

**Keywords:** *Hordeum brevisubulatum*, WRKY group II subfamily, *HbWRKY11*, saline-alkali stress

## Abstract

The WRKY group II subfamily, a major conserved and plant-specific WRKY transcription factor family, plays a central role in regulating plant responses to abiotic stresses. However, systematic characterization of WRKY group II genes and their involvement in saline–alkali stress responses in *Hordeum brevisubulatum* (Trin.) Link remains largely unexplored. In this study, 23 WRKY group II genes were identified at the genome-wide level in *H. brevisubulatum*. Phylogenetic analysis classified these genes into five subgroups, with members within each subgroup exhibiting highly conserved motif compositions and gene structures. Promoter analysis revealed abundant cis-acting elements related to stress and defense responses, phytohormone signaling, growth and development, and light responsiveness, suggesting diverse regulatory potential. Transcriptome sequencing and qRT-PCR analysis showed that several *HbWRKY* genes were responsive to NaHCO_3_-induced saline-alkali stress. Notably, *HbWRKY11* displayed sustained up-regulation and significantly enhanced yeast tolerance to NaHCO_3_ stress. Overall, this study provides the first systematic analysis of WRKY group II genes transcription factors in *H. brevisubulatum* and identifies *HbWRKY11* as a key candidate gene contributing to saline–alkali stress tolerance.

## 1. Introduction

The grass family (Poaceae) is a widely spread plant family with economic and ecological significance, providing staple food, forage and bioenergy resources, covering approximately 40% of the earth’s land surface [1,2]. Members of Poaceae exhibit remarkable adaptability to diverse and extreme environments, which has contributed to their wide geographic distribution [2]. Within Poaceae, the genus *Hordeum* comprises both cultivated barley (*Hordeum vulgare* L.) and several wild relatives that harbor rich genetic diversity for abiotic stress tolerance [3]. These wild *Hordeum* species represent invaluable genetic resources for understanding stress adaptation mechanisms and for improving stress resilience in cereal crops.

Recent advances in genome sequencing technologies have greatly facilitated functional genomics studies in the *Hordeum* genus. A high-quality reference genome of *Hordeum marinum* Huds., a halophytic wild barley species, was released in 2022, providing novel insights into salt tolerance mechanisms in barley relatives [4]. More recently, the chromosome-scale genome assembly of *H. brevisubulatum* was published in 2025 [5]. These newly available genomic resources have enabled systematic identification and comparative analysis of gene families. As an illustration, *StCPAI* gene family in potato and *ALOG* gene family in rice have been analyzed with such genome statistics [6,7]. However, functional studies of stress-related transcription factors in wild *Hordeum* species, particularly *H. brevisubulatum*, remain poorly understood [8].

Soil salinization is among the most severe environmental constraints threatening global agricultural productivity. Excessive saline-alkali conditions disrupt plant water uptake, ion homeostasis, and metabolic processes, ultimately leading to growth inhibition and yield loss [9]. It is estimated that more than 7% of total land worldwide is affected by salt stress, and the affected area continues to expand due to climate change and unsustainable land-use practices [10]. Therefore, identifying stress-resilient plant species and uncovering key genes conferring saline-alkali tolerance is of great importance for both ecological restoration of degraded lands and genetic improvement of crops.

*Hordeum brevisubulatum* is a perennial grass species naturally distributed in saline-alkali grasslands of northern Central Asia [11]. It exhibits exceptional tolerance to saline-alkali stresses, and can survive and reproduce under extreme soil conditions that are lethal to most crops [5]. Similarly, *H. marinum* is adapted to coastal and inland saline environments and has long been recognized as a valuable genetic resource for salt tolerance [4]. Cultivated barley (*H. vulgare*), although less tolerant than its wild relatives, is considered one of the most salt-tolerant cereal crops among major grasses [12]. The coexistence of cultivated and halophytic species within the *Hordeum* genus provides an ideal system for comparative genomic and functional studies aimed at identifying key stress-adaptive genes.

Transcription factors play central roles in regulating plant responses to multiple biological process including environmental stresses, by modulating the expression of target genes [13], among which the WRKY transcription factor family is one of the largest plant-specific transcription factor families and is characterized by the highly conserved WRKY domain and a zinc finger motif [12,14]. WRKY proteins are widely involved in plant growth and development, hormone signaling, pathogen defense, and responses to abiotic stresses, and they are classified into three major groups (groups I, II, and III), with group II further subdivided into five subgroups (IIa–IIe) based on the number of WRKY domains and the type of zinc finger motif [15]. Notably, group II WRKY transcription factors constitute the largest subgroup and exhibit remarkable functional diversity. Numerous group II *WRKY* genes, like *OsWRKY72*, *PgWRKY44* and *TaWRKY17*, have been reported to enhance salt tolerance [16,17,18]. All these reports suggest that group II WRKY transcription factors often function as key nodes in complex regulatory networks governing plant abiotic stress adaptation, highlighting their potential as targets for crop genetic improvement.

In recent years, genome-wide analysis of specific species has been broadly applied to researching the potential influence of a particular gene family on environmental stress, among which salt stress is included [19]. Moreover, genome-wide identification and expression profiling of *WRKY* gene families have also been reported in multiple plant species, including *Cucurbita maxima* [20], *Asparagus officinalis* [21], *Ginkgo biloba* [22], *Malus domestica* [23] and etcetera, with increasing attention to group II WRKY members due to their abundance and broad involvement in abiotic stress responses [24]. Although such published studies have systematically characterized WRKY transcription factor families in cereal crops and revealed their regulatory roles in salt, drought and saline-alkali saline-alkali stress tolerance, comprehensive analyses of group II *WRKY* genes in halophytic wild barley species, particularly *H. brevisubulatum*, are still lacking.

In addition to transcriptomic and bioinformatic analyses, heterologous expression systems have been widely used to investigate the functions of stress-responsive genes. Among them, the yeast expression system provides a convenient and efficient platform for evaluating the roles of candidate genes in stress tolerance due to its genetic tractability and well-characterized stress response pathways [25]. Previous studies have demonstrated that heterologous expression of plant transcription factors in yeast can enhance tolerance to various abiotic stresses. For example, the transcription factor *LcWRKY40* significantly enhanced tolerance to saline–alkali stress in yeast cells [26]. These findings indicate that heterologous expression in yeast can provide valuable preliminary evidence for the involvement of plant transcription factors in abiotic stress responses.

In this study, we performed a genome-wide identification of group II WRKY transcription factors in *H. brevisubulatum* and comparative analysis between *H. brevisubulatum, H. marinum* and *H. vulgare*. Focusing on *H. brevisubulatum*, we systematically analyzed the phylogenetic relationships, gene structures, conserved motifs, cis-acting regulatory elements, collinearity relationships, and expression patterns of group II *WRKY* genes. Through integrative analyses, we identified several candidate group II *WRKY* genes potentially involved in saline-alkali stress tolerance, providing a solid foundation for subsequent functional validation and offering valuable candidate genes for the genetic improvement of stress tolerance in cereal crops.

## 2. Results

### 2.1. Phylogenetic and Physicochemical Properties Analyses of Group II HbWRKYs

**Figure 1:**
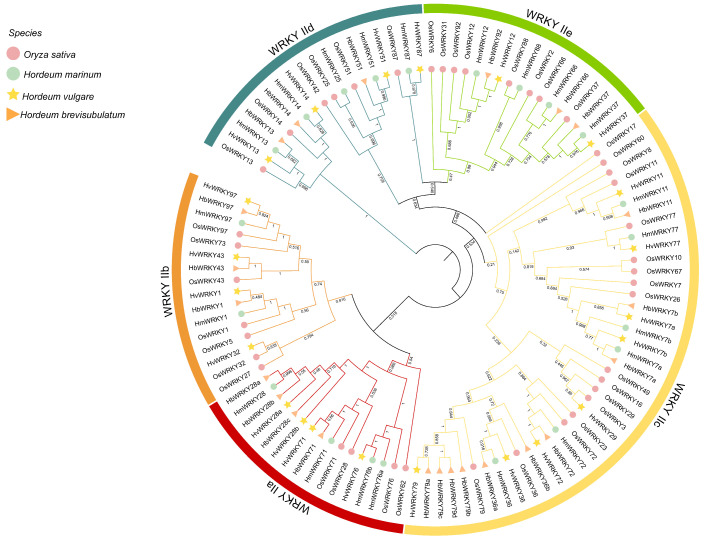
Phylogenetic tree of group II WRKY members from *H. brevisubulatum*, *H. marinum*, *H. vulgare*, and rice (*O. sativa*). The amino acid sequences of HbWRKYs were marked with orange triangles; green circles for HmWRKYs; yellow stars for HvWRKYs and pink circles for OsWRKYs. The numbers at the branches represent bootstrap support values.

Phylogenetic analysis was performed to investigate the evolutionary relationships and classifications of the *H. brevisubulatum* group II *WRKY* subfamily proteins. Using BLASTp searches implemented in TBtools v2.390, we identified WRKY group II proteins in *H. brevisubulatum*, *H. marinum*, *H. vulgare*, and rice (*Oryza sativa* L.) (Table S1). A phylogenetic tree was constructed using MEGA7.0 based on full-length amino acid sequences (Figure 1). The group II WRKY members were classified into five subgroups, namely IIa, IIb, IIc, IId and IIe. Among them, subgroup IIc contains the largest number of members, including ten HbWRKYs, eight HvWRKYs, six HmWRKYs and seventeen OsWRKYs. In addition, WRKY11, WRKY7, WRKY36 and WRKY72 formed conserved orthologous sets shared by all four species, suggesting strong evolutionary conservation of these members across *Hordeum* species.

To further characterize group II WRKY proteins in the three *Hordeum* species, we evaluated key physicochemical properties, including protein length, molecular weight (MW), theoretical isoelectric point (pI) and instability index (Table S2). Group II WRKY proteins ranged from 187 to 595 amino acids in length, indicating substantial variation in protein size. Consistently, predicted MW values varied from 20,935.54 to 61,196.21 Da. Theoretical pI values ranged from 5.08 to 10.08, suggesting that group II WRKY proteins include both acidic and basic members. Moreover, the average grand average of hydropathicity (GRAVY) values for group II WRKY proteins in all three *Hordeum* species are negative, indicating that these proteins are predominantly hydrophilic.

### 2.2. Chromosomal Distribution of Group II WRKY Genes

**Figure 2:**
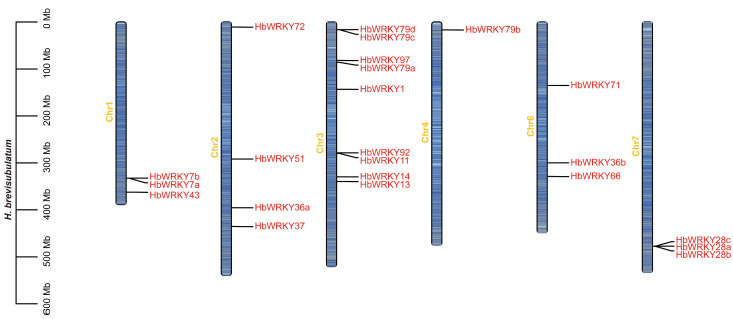
Chromosomal location of group II *WRKY* genes in *H. brevisubulatum*.

Following the phylogenetic classification, we mapped group II *WRKY* genes to chromosomes in *H. brevisubulatum* and compared the distribution with *H. vulgare* and *H. marinum* (Figure 2 and Figure S1). In *H. brevisubulatum*, 23 group II *WRKY* loci were unevenly distributed across six chromosomes, with nine genes on chromosome 3, four on chromosome 2, three on chromosomes 1, six and seven and one on chromosome 4 (Figure 2).

### 2.3. Gene Structure and Motif Analysis of Group II WRKY Genes in H. brevisubulatum

**Figure 3:**
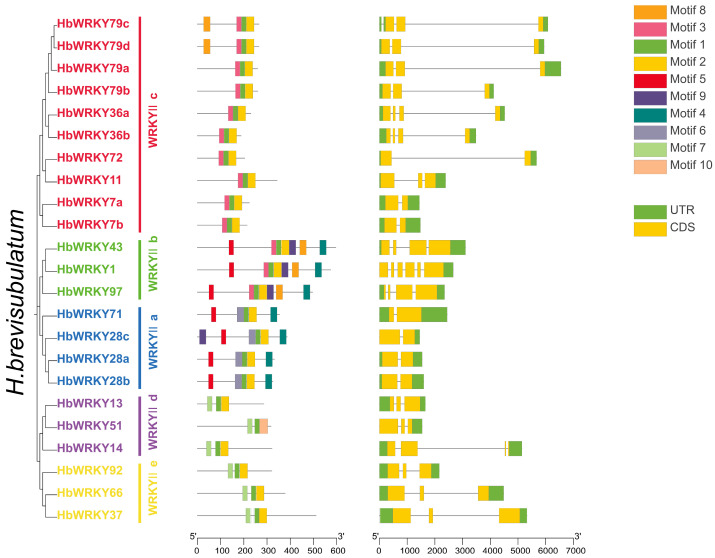
Conserved motifs and exon–intron structures of group II *WRKY* genes in *H. brevisubulatum*. The members listed are categorized by group II *WRKY* subfamilies they belongs to.

The conserved protein features and gene structures are further examined using motif and exon–intron analyses (Figure 3 and Figure S2). Simultaneously, we conducted motif analysis to characterize conserved structural features of group II WRKY proteins for *H. brevisubulatum*, *H. marinum*, and *H. vulgare* by MEME. Ten conserved motifs were identified and HbWRKY proteins demonstrate strong motif conservation and members within the same phylogenetic subclade generally share similar motif compositions and arrangements (Figure 3). Notably, Motif 1 and Motif 2 are consistently located toward the C-terminus of most HbWRKY proteins and highly position-conserved, supporting their correspondence to the core WRKY DNA-binding domain. Subclade IIc members typically display a compact motif organization dominated by Motif 1–3, whereas IIa and IIb members tend to contain additional motifs, indicating greater structural complexity. Although IId and IIe include fewer members in *H. brevisubulatum*, their motif patterns are relatively stable within each subclade. As an illustration, IId proteins commonly contain Motif 7 and the motif landscape presents a conserved pattern across species. Orthologous group II *WRKY* sets among *H. brevisubulatum*, *H. marinum* and *H. vulgare* generally retained highly similar motif compositions and orders (Figure S2), indicating that the overall protein structural framework of group II *WRKY*s is conserved across *Hordeum* species. Such conservation is particularly evident for IIc orthologs, which consistently exhibit streamlined motif architectures in all three species.

To assess gene-structure evolution, motif organization is compared with exon-intron architectures (Figure 3). Most group II *WRKY* genes in *H. brevisubulatum* contain annotated 5′ and 3′ untranslated regions (UTRs) and comprise 2–6 exons. Consistent with the motif results, genes within the same subclade also demonstrate conserved exon–intron organizations. The group IIc genes generally contain fewer exons and simpler gene structures matching their compact motif profiles, whereas IIa/IIb genes tend to display more complex architectures with increased exon numbers and more variable intron lengths. Together, these results indicate that diversification among group II WRKY subclades is reflected at both the protein motif level and the gene-structure level.

### 2.4. Cis-Acting Element Analysis of Group II WRKY Genes in H. brevisubulatum

**Figure 4:**
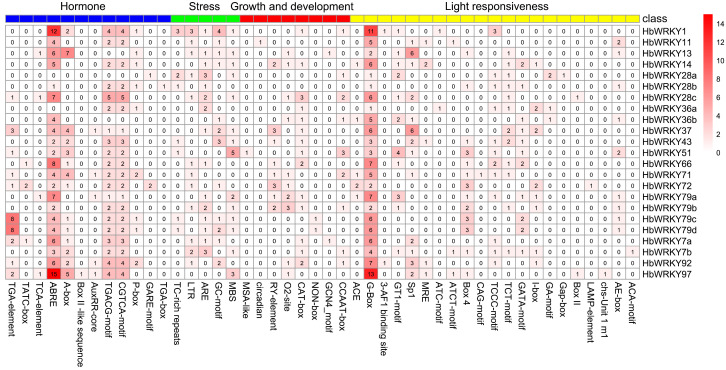
The distribution of cis-acting elements in the promoters of group II *WRKY* genes in *H. brevisubulatum*. Group II *WRKY* genes are shown on the right and cis-acting elements that are classified into four categories (hormone responses, stress responses, growth and development, and light responsiveness) are listed at the bottom. The numbers within each block of the heatmap indicate the count of cis-acting elements present in the corresponding group II *WRKY* gene, with a red color gradient representing element abundance.

To characterize the transcriptional regulatory features of group II *WRKY* genes in *H. brevisubulatum*, we scanned the 2000 bp upstream promoter regions for cis-acting regulatory elements. Four major categories of cis-elements were identified in *HbWRKY* promoters, including elements related to stress and defense responses, phytohormone responsiveness, growth and development, and light responsiveness. Stress-related and defense-related elements, including MBS (drought responsiveness), ARE and GC-motif (anaerobic inducement), LTR (low-temperature responsiveness) and TC-rich repeats are also broadly present across *HbWRKY* promoters, indicating potential involvement in multiple stress-response pathways. Hormone-responsive elements were widely detected, particularly MeJA-responsive motifs (TGACG- and CGTCA-motifs) and ABREs, which were enriched in several promoters such as *HbWRKY1*, *HbWRKY79a*, and *HbWRKY97*. Cis-acting elements associated with growth and development, comprising seed-specific regulation (RY-element), endosperm expression (GCN4-motif), meristem expression (CAT-box), storage protein metabolism (O2-site), and circadian control (circadian), were identified across the three *Hordeum* species (Figure 4 and Figure S3). Notably, circadian-related regulatory elements were detected exclusively in the promoter of *HbWRKY11* and were absent from other *HbWRKY* promoters, suggesting a gene-specific potential for time-dependent transcriptional regulation. Light-responsive elements represent the most abundant category among all identified cis-elements. There are 23 cis-elements detected in *H. vulgare* and 22 in both *H. marinum* and *H. brevisubulatum*, with the G-box being the most frequently identified motif. Comparative analysis with *H. marinum* and *H. vulgare* shows that the major cis-element categories are largely conserved across species, whereas the number and arrangement of specific elements vary among orthologous *WRKY* genes (Figure S3).

### 2.5. Collinearity and Ka/Ks Analyses

**Figure 5:**
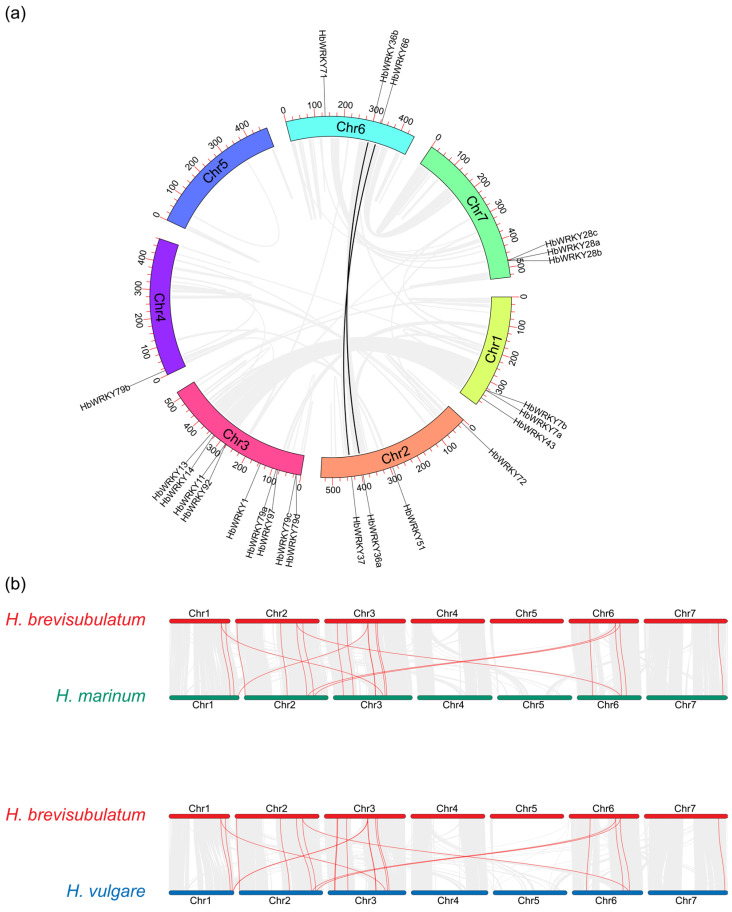
Evolutionary analysis of group II *WRKY* genes in *H. brevisubulatum*. (**a**) Intraspecific collinearity of group II *WRKY* genes within the *H. brevisubulatum* genome. Gray lines indicate genome-wide syntenic blocks and black lines highlight the group II *WRKY* pairs. (**b**) Interspecific collinearity of group II *WRKY* genes between *H. brevisubulatum* and *H. vulgare* (upper) and between *H. brevisubulatum* and *H. marinum* (lower). Red lines represent orthologous *WRKY* gene pairs.

To explore the expansion history and evolutionary conservation of group II *WRKY* genes in *H. brevisubulatum*, we performed intraspecific and interspecific collinearity analysis. Within the *H. brevisubulatum* genome, two syntenic group II *WRKY* gene pairs were detected, which are located on different chromosomes (Figure 5a). Such a distribution pattern indicates that group II *WRKY* expansion in *H. brevisubulatum* is mainly attributable to segmental and ancient whole-genome duplication, rather than recent tandem duplication. Afterwards, we examined the evolutionary conservation across different *Hordeum* species (Figure 5b). It was shown that there were 23 one-to-one syntenic orthologous group II *WRKY* gene pairs between *H. brevisubulatum* and *H. vulgare* (Table S4) and 21 orthologous pairs between *H. brevisubulatum* and *H. marinum* (Table S5), supporting a largely conserved chromosomal framework. Furthermore, we estimated the Ka/Ks for each gene, demonstrating that all these members represent Ka/Ks < 1 (Figure S4), indicating that group II *WRKY* genes have been mainly maintained under purifying selection throughout evolution.

### 2.6. Expression Patterns of H. brevisubulatum Group II WRKY Genes Under Saline-Alkali Stress

**Figure 6:**
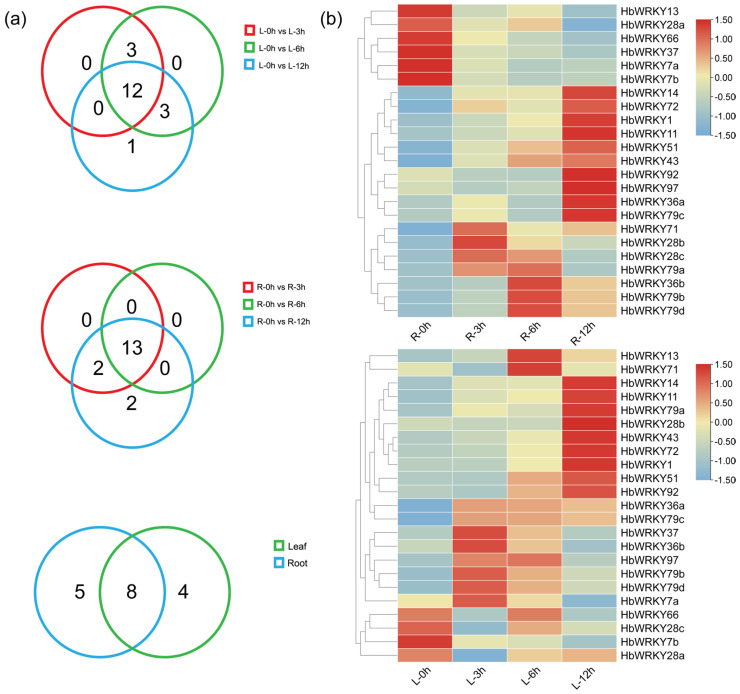
Identification of salt–alkali responsiveness of group II *WRKY* genes in *H. brevisubulatum* based on transcriptome. (**a**) Venn diagrams displaying the numbers of group II *WRKY* genes up-regulated at 3, 6, and 12 h compared with 0 h under 200 mM NaHCO_3_ treatment in leaves (upper), roots (middle) and the overlap between leaf and root datasets (lower). (**b**) Heatmap of standardized expression levels (FPKM) of group II *WRKY* genes in leaves (upper) and roots (lower) at 0, 3, 6 and 12 h under NaHCO_3_ treatment. Genes were hierarchically clustered based on expression patterns. The color scale represents the relative expression levels of genes after standardization. The values displayed are log2-transformed fold changes, where positive values indicate upregulation, and negative values indicate downregulation relative to the baseline sample.

To identify the group II *WRKY* genes in *H. brevisubulatum* that potentially respond to saline-alkali stress, we examined the expression patterns of the group II *WRKY* genes in roots and leaves of 13-day-old *H. brevisubulatum* seedlings under 200 mM NaHCO_3_ treatment at 0, 3, 6 and 12 h by transcriptome sequencing. As shown in the Venn diagram (Figure 6a), compared with 0 h, 12 genes in leaves and 13 genes in roots were up-regulated at 3, 6, and 12 h, respectively, under saline–alkali stress. Intersection analysis of these gene sets identified eight candidate genes (*HbWRKY11*, *HbWRKY14*, *HbWRKY43*, *HbWRKY1*, *HbWRKY79a*, *HbWRKY79b*, *HbWRKY79d* and *HbWRKY72*) that were consistently up-regulated in both tissues at all three time points. Among these, the expression of *HbWRKY11*, *HbWRKY14*, *HbWRKY43*, and *HbWRKY1* increased progressively with the duration of stress, indicating a time-dependent inducement pattern.

### 2.7. Expression Patterns of HbWRKYs Under Abiotic Stresses

**Figure 7:**
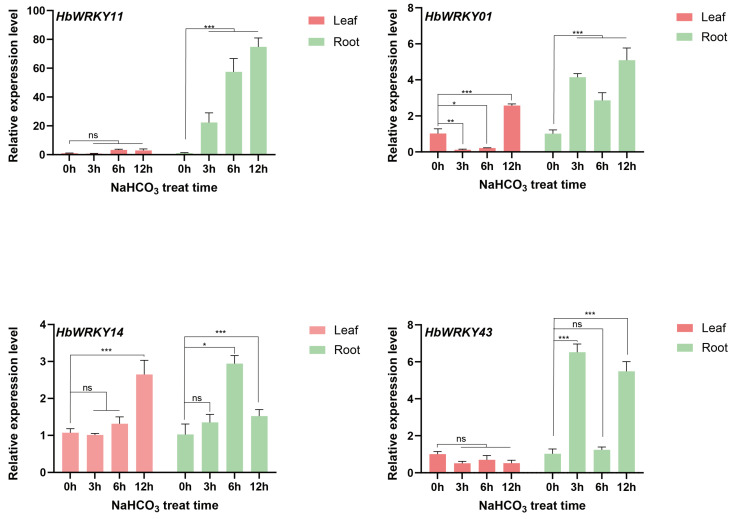
qRT-PCR analysis of *HbWRKY11*, *HbWRKY1*, *HbWRKY14*, and *HbWRKY43* in leaves and roots of *H. brevisubulatum* under 200 mM NaHCO_3_ treatment at 0, 3, 6, and 12 h. Error bars represent the standard error of three biological replicates and asterisks indicate a significant difference between the treatment and 0 h group (* *p* < 0.05; ** *p* < 0.01; *** *p* < 0.001; ns means not significant.).

A qRT-PCR analysis was performed to validate the expression patterns of four candidate genes (*HbWRKY11*, *HbWRKY1*, *HbWRKY14*, and *HbWRKY43*) identified from the RNA-seq–based intersection screening (Figure 7). Overall, the qRT-PCR results supported the presence of distinct tissue- dependent and time-dependent inducement patterns under 200 mM NaHCO_3_ treatment. *HbWRKY11* exhibits minimal expression changes in leaves throughout the treatment period, whereas in roots it displays a clear time-dependent inducement, with transcription levels increasing markedly at 6 h and further rising at 12 h. This progressive inducement is accompanied by the largest expression amplitude among the four genes, highlighting a strong and sustained root-specific response. *HbWRKY1* shows limited inducement in leaves, with an increase detectable only at the late stage. In contrast, its expression in roots can be rapidly and strongly induced, showing pronounced up-regulation from 3 h onward and remaining elevated at later time points. For *HbWRKY14*, expression levels in leaves remained relatively stable across all time points, while in roots, a transient inducement is observed at 6 h, followed by a return toward basal levels. *HbWRKY43* displays no obvious expression changes in leaves but a distinct root-specific response, characterized by strong inducement at early and late stages of treatment with little change at the intermediate time point. Taken together, integration of the RNA-seq screening and qRT-PCR validation indicates that stress-induced transcriptional responses of these group II *WRKY* genes are predominantly manifested in roots. Among them, *HbWRKY11* is distinguished by its sustained and high-magnitude inducement during prolonged stress exposure, thereby emerging as the most prominent candidate for subsequent functional characterization.

### 2.8. The Potential of HbWRKY11 Conferring Saline-Alkali Stress Tolerance

**Figure 8:**
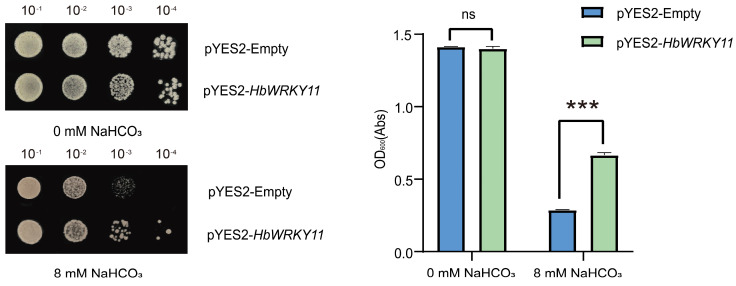
*HbWRKY11* enhances yeast tolerance to NaHCO_3_ stress. Growth of yeast strains carrying the empty vector (pYES2-Empty) or expressing *HbWRKY11* under control (0 mM) and NaHCO_3_ (8 mM) conditions. (**Left**), OD_600_ measurements of yeast cultures (mean ± SD, *n* = 3). (**Right**), serial dilution spot assays (10^−1^–10^−4^) on SD/−Ura medium. Statistical significance was determined by Student’s t-test; ns, not significant; ***, *p* < 0.001.

To assess the contribution of *HbWRKY11* to saline-alkali stress tolerance, we compared the yeast strains carrying pYES2-*HbWRKY11* with those carrying an empty vector under control and NaHCO_3_ treatment conditions (Figure 8). The transformed yeast strains were confirmed by colony PCR prior to the stress tolerance assays (Figure S5). Under non-stressed conditions (0 mM NaHCO_3_), the two strains exhibit comparable growth patterns in the serial dilution assay, and no evident difference was observed in OD_600_ values, indicating that *HbWRKY11* expression did not affect basal yeast growth. In contrast, under 8 mM NaHCO_3_, clear differences in growth performance were detected. The empty-vector control presents markedly reduced growth, particularly at higher dilution levels, whereas the strain with *HbWRKY11* maintained stronger colony formation across the dilution series. Consistent with the spot assay, OD_600_ measurements revealed that the strain carrying *HbWRKY11* forms a colony with significantly higher cell density under saline-alkali stress conditions compared with the control group. Together, the concordant phenotypes observed in both the spot assay and OD_600_ quantification indicate that heterologous expression of *HbWRKY11* enhances yeast tolerance to NaHCO_3_-induced saline-alkali stress, supporting its functional involvement in stress adaptation.

## 3. Discussion

Saline-alkali stress is rapidly expanding worldwide, and the combined effects of salt stress and high pH conditions can simultaneously induce ionic toxicity, osmotic and oxidative stress, thus severely inhibiting plant growth and reducing crop productivity [9]. Under this context, exploring genes with saline-alkali tolerance is not only essential for the ecological restoration of saline–alkali lands but also provides an important molecular basis for the development of saline-alkali tolerant crop varieties for food security. *Hordeum brevisubulatum* is a dominant plant species in saline–alkali soils and exhibits strong tolerance to saline-alkali stress [5,27]. Owing to these characteristics, it represents an ideal material for elucidating the molecular mechanisms underlying saline–alkali tolerance and for identifying candidate genes regulating saline-alkali stress. *WRKY* transcription factors constitute one of the core regulatory families involved in plant stress responses and represent a large transcription factor family in plants [28,29]. Members of this family play critical roles in plant growth and development, phytohormone signaling, and responses to various abiotic stresses [30,31]. Although the *WRKY* gene family has been systematically identified in many plant species, including halophytic and stress-adapted taxa, studies focusing on specific WRKY subgroups in *H. brevisubulatum* remain limited [32,33].

In this study, a total of 23 group II *WRKY* genes were identified in *H. brevisubulatum*. Physicochemical analyses showed that the 23 group II WRKY proteins identified in *H. brevisubulatum* varied considerably in amino acid length, molecular weight, and theoretical isoelectric point values above 7, suggesting a predominance of basic amino acids within this subfamily (Table S2). This feature is consistent with observations reported for WRKY proteins in monocot species [34], but differs from patterns reported in some dicot species [35], indicating potential divergence of WRKY family characteristics during monocot–dicot evolution. Phylogenetic analysis classified group II WRKY members from *H. brevisubulatum*, *H. vulgare* and *H. marinum* into five subgroups (IIa–IIe), with the IIc subgroup representing the largest clade in all three species (Figure 1). This pattern is consistent with previous reports of IIc expansion in rice and other monocots [30], suggesting that this subgroup has been evolutionarily conserved and may retain important biological functions. Chromosomal mapping further revealed that group II *WRKY* genes in *H. brevisubulatum* were unevenly distributed across chromosomes. The largest number of genes was located on chromosome 3, whereas no group II *WRKY* genes were detected on certain chromosomes (Figure 2 and Figure S1). Comparative analysis with *H. vulgare* and *H. marinum* showed that although the overall chromosomal framework of group II *WRKY* genes was largely conserved within the genus *Hordeum*, notable differences existed in gene number and chromosomal localization. These differences likely reflect lineage-specific gene duplication, rearrangement, or loss events during species divergence [36,37,38].

Despite substantial variation in physicochemical properties, group II *WRKY* genes in *H. brevisubulatum* exhibited relatively conserved gene structures and motif compositions (Figure 3). The 23 identified group II *WRKY* genes were distributed with abundant CDS and intron regions, and it has been shown that the higher the number of introns, the longer the gene sequences and the higher the recombination frequency among the genes [39]. Most HbWRKY proteins contained Motif 1 and Motif 2 at the C-terminus, corresponding to the conserved WRKY DNA-binding domain. Distinct structural differences were observed among subgroups, with members of the IIc subgroup typically containing only Motif 1-Motif 3 and possessing fewer exons, resulting in compact gene structures, whereas IIa and IIb subgroup members harbored additional motifs and exhibited more complex exon–intron organizations. Such structural variation may provide a molecular basis for functional diversification among different group II WRKY subgroups. Promoter cis-element analysis further provided insights into potential regulatory mechanisms (Figure 4). Cis-acting elements in promoters play a pivotal role in regulating transcription, exerting a direct influence on the levels and biological roles of gene product [40]. Stress and defense-related elements closely associated with saline–alkali stress were detected in multiple *HbWRKY* promoters, suggesting that these members may be involved in typical stress signaling pathways. In addition, MeJA and ABA responsive elements were also enriched, suggesting that group II *WRKY* genes may participate in the integration of hormone and stress signaling pathways. Importantly, circadian-related regulatory elements were detected exclusively in the promoter of *HbWRKY11* among group II *WRKY* genes in *H. brevisubulatum*, indicating a gene-specific potential for time-dependent transcriptional regulation.

Gene duplication is one of the primary driving forces underlying the expansion of plant gene families, with segmental duplication and tandem duplication representing the two major forms of gene duplication [41,42]. It was found that 124 gene duplication events were identified in the *Trifolium repens WRKY* gene family, including 118 segmental duplications and 6 tandem duplications [43]. In *Platostoma palustre*, a total of 116 gene duplication events were identified, including 112 segmental duplications and 4 tandem duplications [44]. Group II *WRKY* genes contained 2 segmental duplication genes and did not involve tandem duplications (Figure 5a). It was similar to the previous results, suggesting that the expansion of the group II *WRKY* gene subfamily may be primarily driven by segmental duplications. Collinearity was more extensive between *H. brevisubulatum* and *H. vulgare* than between *H. brevisubulatum* and *H. marinum* (Figure 5b), indicating a closer evolutionary relationship with cultivated barley. Together with Ka/Ks ratios predominantly below 1, these results suggest that group II *WRKY* genes are functionally conserved and have undergone purifying selection. Therefore, key candidates identified in *H. brevisubulatum* represent promising candidate genes for breeding saline-alkali tolerant barley and other cereal crops.

Further transcriptome analysis under 200 mM NaHCO_3_ treatment revealed that group II *WRKY* genes exhibit pronounced temporal and tissue-specific expression patterns in both roots and leaves. Through multi-time-point intersection screening, a subset of genes showing sustained up-regulation in both tissues was identified, suggesting their potential involvement in core regulatory processes underlying saline–alkali stress responses. Subsequent qRT-PCR validation further refined these expression patterns and showed that, among the candidate genes, *HbWRKY11* displayed a progressively enhanced expression in roots as stress duration increased, with a markedly higher expression amplitude than the other candidates. Given that roots are the primary organs for early perception and response to saline–alkali stress, together with the presence of a circadian-related cis-regulatory element uniquely detected in the promoter of *HbWRKY11*, this gene is likely involved in time-dependent transcriptional regulation during saline–alkali stress. Notably, *Hb*WRKY*11* is orthologous to *OsWRKY11* in rice. Previous studies have demonstrated that *OsWRKY11* is a typical stress-inducible WRKY transcription factor that is strongly induced by heat shock and drought stress in rice seedlings [45]. Overexpression of *OsWRKY11* driven by the rice *HSP101* promoter significantly enhanced tolerance to both heat and drought stress, as evidenced by delayed leaf wilting, increased survival of green tissues, and reduced water loss from detached leaves [45]. These findings indicate that *OsWRKY11* functions as a positive regulator in multiple abiotic stress responses. Also, *HbWRKY11* exhibited higher expression levels in roots under saline–alkali stress, suggesting that it may not only retain a conserved stress-regulatory function similar to that of its rice ortholog, but also play an additional role in mediating saline–alkali stress responses, thereby contributing to stress adaptation in *H. brevisubulatum*. To further evaluate the regulatory potential of *HbWRKY11* under saline–alkali stress, its function was preliminarily examined using a heterologous yeast expression system. Heterologous expression of *HbWRKY11* did not affect basal yeast growth under non-stress conditions but significantly enhanced growth under NaHCO_3_ stress. This phenotype was consistently observed in both serial dilution spot assays and OD_600_-based quantitative measurements. Together with the transcriptomic and qRT-PCR data, these results support a positive role of *HbWRKY11* in saline–alkali stress tolerance and suggest that it represents an important member of the group II *WRKY* family involved in stress adaptation in *H. brevisubulatum*. Future studies in plant systems are required to elucidate the downstream regulatory network of *HbWRKY11* and its specific roles in ion homeostasis and stress signaling pathways, thereby providing a foundation for the identification of genes involved in saline-alkali tolerance and their potential application in economically important crops.

## 4. Materials and Methods

### 4.1. Identification of Group II WRKY Genes in Three Hordeum Species

The *H. brevisubulatum* (GWH database, PRJCA019121 [5]. *H*. *vulgare* (http://doi.org/10.5447/ipk/2021/3 on 6 December 2024) and *H. marinum* (Genome WareHouse (GWH) database, PRJCA009391) [4]. Protein sequences of rice group II *WRKY* genes were collected from previously published studies [30] and verified for the presence of the conserved WRKY domain using the CD-Search database (https://www.ncbi.nlm.nih.gov/Structure/cdd/cdd.shtml accessed on 7 December 2024) [46,47]. These validated rice WRKY proteins were used as queries to identify homologous group II *WRKY* genes in *H. brevisubulatum*, *H. vulgare*, and *H. marinum* by BLASTp searches utilizing TBtools v2.390, with an E-value cut off of 1 × 10^−10^. All candidate proteins were further confirmed by CD-Search to ensure the presence of an intact WRKY domain.

### 4.2. Phylogenetic Analysis and Physicochemical Characterization of the Group II WRKYs

Multiple sequence alignment of the protein sequences of group II *WRKY* genes from *H. brevisubulatum* and the other three species was performed using ClustalW integrated in MEGA7.0 [48]. A phylogenetic tree was constructed using a maximum likelihood method with a bootstrap value set to 1000 replicates. Then, the phylogenetic tree was annotated using iTOL online tools (https://itol.embl.de/ accessed on 3 April, 2025) [49]. The length, molecular mass, theoretical isoelectric points (PI), instability index, and grand average of hydropathy (GRAVY) of group II WRKY proteins were calculated using the Expasy—protparam tool (https://web.expasy.org/protparam/ accessed on 5 April 2025) [50].

### 4.3. Chromosomal Distribution

The chromosomal locations of the group II *WRKY* genes from the three species were obtained from the corresponding General Feature Format (gff) files. The density of genes on the chromosomes was calculated using TBtools v2.390.

### 4.4. Gene Structure Analysis

The conserved motifs of group II *WRKY* genes from *H. brevisubulatum*, *H. vulgare* and *H. marinum* were analyzed using the MEME Wrapper function in TBtools v2.390. The exon/intron structure of each group II *WRKY* gene was visualized using the Gene Structure View function of TBtools v2.390.

### 4.5. Cis-Acting Element Analysis

The upstream 2000 bp sequences of the coding sequences (CDS) of group II *WRKY* genes from *H. brevisubulatum* and the other three species were extracted using TBtools v2.390, and the results were predicted using the PlantCare website (http://bioinformatics.psb.ugent.be/webtools/plantcare/html/ accessed on 19 June 2025) [51]. Different known homeopathic elements were identified using bioinformatics and classified into four categories (phytohormone responsiveness, stress and defense responses, growth and development, and light responsiveness) according to their regulatory function and visualized through R language.

### 4.6. Collinearity Analysis and Ka/Ks Analysis

Intra- and interspecific collinearity of *WRKY* genes was analyzed using MCScanX implemented in TBtools v2.390 [52,53], based on genome assemblies and chromosomal position information. Collinear *WRKY* gene pairs within *H. brevisubulatum* and between *H. brevisubulatum*, *H. vulgare*, and *H. marinum* were identified and visualized using TBtools v2.390. Ka/Ks analysis was generated using R-4.4.2 for Windows software with the ggplot2 (v4.0.1), dplyr (v1.1.4), tidyr (v1.3.1) packages.

### 4.7. Transcriptome Analysis of Group II WRKY Genes in H. brevisubulatum

In this study, Hordeum brevisubulatum was collected from wild populations growing in saline–alkali soils in Anda City, Heilongjiang Province, China (124°53′–125°55′ E, 46°01′–47°01′ N), and seeds were stored at Northeast Forestry University. These seeds were sterilized in 75% ethanol for 5 min, repeated three times. After using sterilized water, wash the seeds 8–10 times. Seeds were sown on filter paper and kept under long-day conditions (16 h light/8 h dark) at 22 °C. After seed germination, uniform seedlings were transferred to containers with 1/4 strength Hoagland’s nutrient solution for 13 days. The 13-day-old seedlings of *H. brevisubulatum* were then transferred to 200 mM NaHCO_3_ for durations of 3, 6, and 12 h; seedlings collected immediately before NaHCO_3_ treatment were used as the 0 h control. Each treatment included at least 40 seedlings and was conducted with three biological replicates. To explore the expression patterns of *HbWRKY* genes, leaves and roots from 13-day-old seedlings of *H. brevisubulatum* were collected after treatment with 200 mM NaHCO_3_ for 0, 3, 6, and 12 h, with three independent biological replicates for each time point. Total RNA was extracted using the Plant RNA Purification Reagent (Invitrogen, Carlsbad, CA, USA). RNA-seq data were deposited in the Genome Warehouse database at the China National Genomics Data Center under BioProject accession number PRJCA042803. Gene expression levels were quantified as Fragments Per Kilobase of transcript per Million mapped reads (FPKM) values and normalized for subsequent analyses (Table S6). Differential expression analysis was performed using DESeq2 [54] on raw count data obtained from RNA-seq. Genes with low counts were filtered prior to analysis. Differential expression between treatment and control conditions was assessed for each time point and tissue separately, with significance determined by an adjusted *p*-value (padj) < 0.05 and log2 fold change ≥ 1 [55]. Expression profiles of group II WRKY genes were visualized using heatmaps generated with TBtools v2.390 to illustrate tissue-specific and temporal expression patterns. Venn diagrams showing overlaps of differentially expressed group II WRKY genes among different time points and tissues were generated in R-4.4.2 using the VennDiagram package (v1.8.2).

### 4.8. Total RNA Extraction, Reverse Transcription, and qRT-PCR Analysis

The 13-day-old seedlings of *H. brevisubulatum* were treated with 200 mM NaHCO_3_ (pH 9.0) for 3, 6 and 12 h. After treatment, roots and leaves were collected for RNA extraction using RNAprep Pure Plant Kit (DP441, Tiangen Biotech, Beijing, China). The RNA concentrations were measured using NanoDrop 2000 spectrophotometer (Thermo Fisher Scientific, Wilmington, DE, USA). The cDNA was synthesized using the TransScript^®^ One-Step gDNA Removal and cDNA Synthesis SuperMix Kit (AT311, TransGen Biotech, Beijing, China) in accordance with the manufacturer’s instructions. Primers for qRT-PCR were designed using Primer Premier 6.0 (Table S7). qRT-PCR was performed according to the protocol using TransStart^®^ Top Green qPCR SuperMix (AQ131, TransGen Biotech, Beijing, China), and three biological replicates were used for each experiment. Relative fold differences were calculated using the 2^−ΔΔCt^ method with *CYP2* (Table S7) as an internal reference gene.

### 4.9. Cloning and Vector Construction of HbWRKY11

Gene-specific amplification primers for *HbWRKY11* were designed using Primer Premier 6.0. The CDS sequence of *HbWRKY11* was amplified using cDNA from root of the two-leaf seedlings of *H. brevisubulatum* as the template. The PCR reaction mixture (50 μL) contained 1 μL of cDNA, 1.5 μL each of primers *HbWRKY11*-F and *HbWRKY11*-R, 25 μL of Prime STAR^®^ Max DNA Polymerase, and 21 μL of ddH_2_O. The PCR program was as follows: 95 °C for 5 min; 35 cycles of 95 °C for 30 s, 53.9 °C for 10 s, and 68 °C for 2 min; followed by a final extension at 68 °C for 5 min and holding at 4 °C. The PCR products were verified by agarose gel electrophoresis and DNA sequencing to obtain the confirmed *HbWRKY11* CDS sequence. Appropriate homologous arms were integrated at the ends of the specific amplification primers targeting the multiple cloning site. The amplified *HbWRKY11* sequence was then used to construct the pYES2-*HbWRKY11* vector. Positive yeast transformants were further verified by colony PCR using gene-specific primers. The primers used are listed in Table S8.

### 4.10. Phenotypic Analysis of saline-alkali Tolerance in Yeast

The *Saccharomyces cerevisiae* competent strain INVSc1 was purchased from Invitrogen and used for yeast expression experiments. The yeast expression vector pYES2 was used and the primers used are listed in Table S8. For plate preparation, filter-sterilized NaHCO_3_ (final concentration: 8 mM) was added to the autoclaved medium after cooling to approximately 50 °C. Wild-type and transgenic yeast strains were streaked onto freshly prepared SD/-Ura solid medium and incubated at 30 °C for 2 days. Single colonies of both the wild-type (Wt) and *HbWRKY11* yeast strains were picked and inoculated into 5 mL of SD/-Ura liquid medium, followed by overnight incubation at 30 °C with shaking at 220 rpm. The yeast cells were collected by centrifugation, and the cell density was adjusted to an OD_600_ between 0.6 and 0.8, ensuring consistent concentrations between the control and transgenic strains. The experimental group (*HbWRKY11* yeast culture) and the control group (empty vector yeast culture) were serially diluted (10^−1^ to 10^−4^). A 3.5 μL aliquot from each dilution was spotted onto the surface of solid medium containing 8 mM NaHCO_3_. The plates were incubated upside down at 30 °C for 3–4 days, and colony growth status was observed every 9 h.

## 5. Conclusions

In this study, a comprehensive genome-wide identification and characterization of group II WRKY transcription factors was conducted in the *H. brevisubulatum*, with comparative analyses across *H. vulgare* and *H. marinum*. We identified 23 group II *WRKY* genes in *H. brevisubulatum* and classified them into five conserved subgroups (IIa–IIe). Transcriptome profiling and qRT-PCR validation demonstrated that several group II *WRKY* genes exhibit pronounced tissue-specific and time-dependent expression patterns under saline-alkali stress, particularly in roots.

Among these, *HbWRKY11* was identified as a prominent candidate due to its sustained and strong inducement during prolonged NaHCO_3_ treatment and the presence of unique circadian-related cis-regulatory elements in its promoter. Furthermore, its conserved ortholog in rice, *OsWRKY11*, also exhibits stress-related responsiveness [51]. Yeast strain expression system further confirmed that *HbWRKY11* enhances tolerance to saline-alkali stress, supporting its positive regulatory role in stress adaptation.

Overall, this study provides the first systematic insight into the group II WRKY transcription factor family in *H. brevisubulatum* and highlights *HbWRKY11* as a key candidate gene associated with saline-alkali tolerance stress. These findings not only deepen our understanding of the molecular mechanisms underlying stress adaptation in *H. brevisubulatum* but also offer valuable genetic resources and candidate genes for the improvement of saline-alkali tolerance in cultivated barley and other cereal crops.

## Data Availability

Data are contained within the article and Supplementary Materials.

## Supplementary Materials

The following supporting information can be downloaded at: https://www.mdpi.com/article/10.3390/plants15060926/s1. Figure S1: Chromosomal location of group II *WRKY* genes in *H. vulgare* and *H. marinum*.; Figure S2: Conserved motifs and exon–intron structures of group II *WRKY* genes in *H. vulgare* and *H. marinum*; Figure S3: The distribution of cis-acting elements in the promoters of group II *WRKY* genes in *H. vulgare* and *H. marinum*; Figure S4: Ka/Ks ratios of orthologous group II *WRKY* gene pairs between *H. brevisubulatum* and *H. marinum*, and between *H. brevisubulatum* and *H.* vulgare; Figure S5: PCR identification of yeast transformants carrying *HbWRKY11*. Table S1: The BLASTp alignment analysis results between the group II WRKY protein sequences of three Hordeum species and those of rice; Table S2: Basic information of group II *WRKY* gene family members; Table S3: Analysis and distribution of conserved motifs in group II WRKY proteins; Table S4: Synteny analysis of *WRKY* genes in *H. brevisubulatum* and *H. vulgare*; Table S5: Synteny analysis of *WRKY* genes in *H. brevisubulatum* and *H. marinum*; Table S6: The expression FPKM of group II *HbWRKY* genes under 200 mM NaHCO_3_ treatment; Table S7: Primers for qRT-PCR; Table S8: Primers for complimentary assay.
