# Supplementary material for: Genome-Wide Identification of WRKY Group II Genes and the Role of HbWRKY11 in Hordeum brevisubulatum Under Saline-Alkali Stress
Source: Plants (Basel). 2026 Mar 17;15(6):926. doi: 10.3390/plants15060926 (PMC13030238; doi:10.3390/plants15060926)
Supplement: Supplementary file 1 [file plants-15-00926-s001.zip › supplementary figures.pdf]

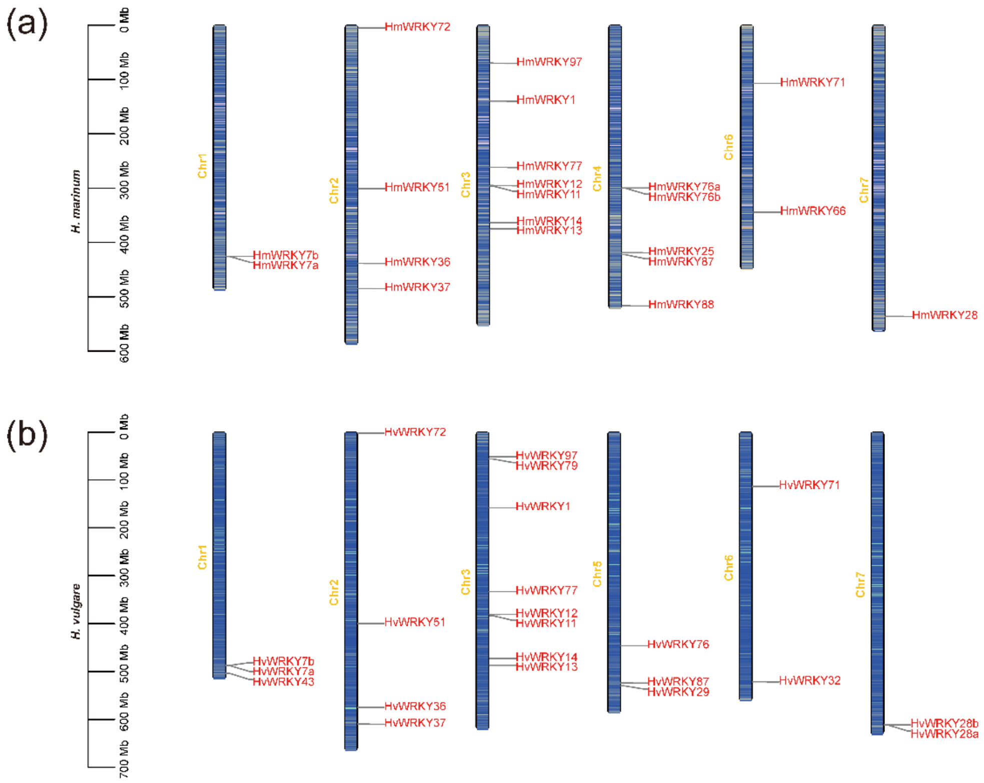

Figure S1. Chromosomal location of Group II *WRKY* genes in *H. vulgare* and *H. marinum*.

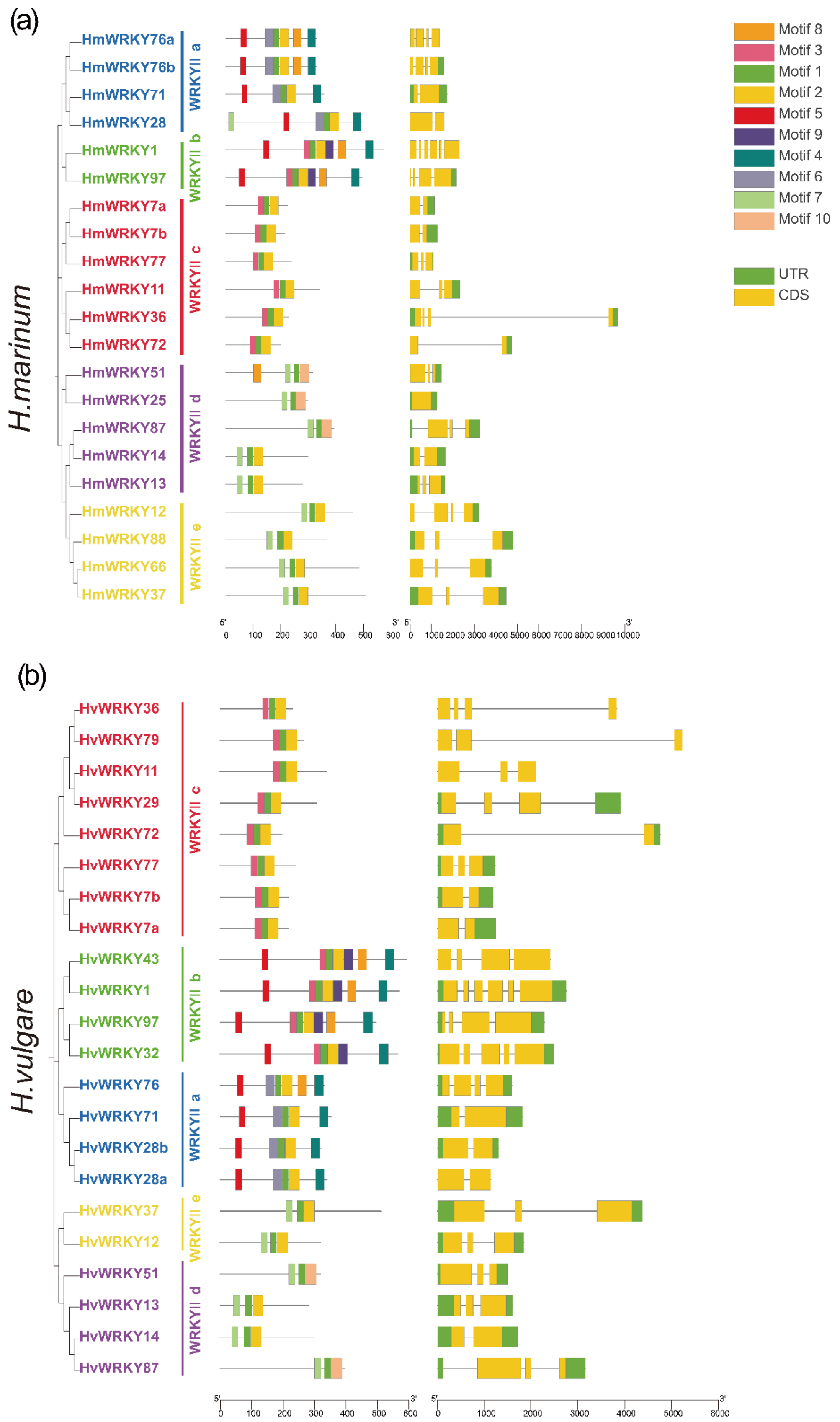

Figure S2. Conserved motifs and exon–intron structures of Group II *WRKY* genes in *H. vulgare* and *H. marinum*.

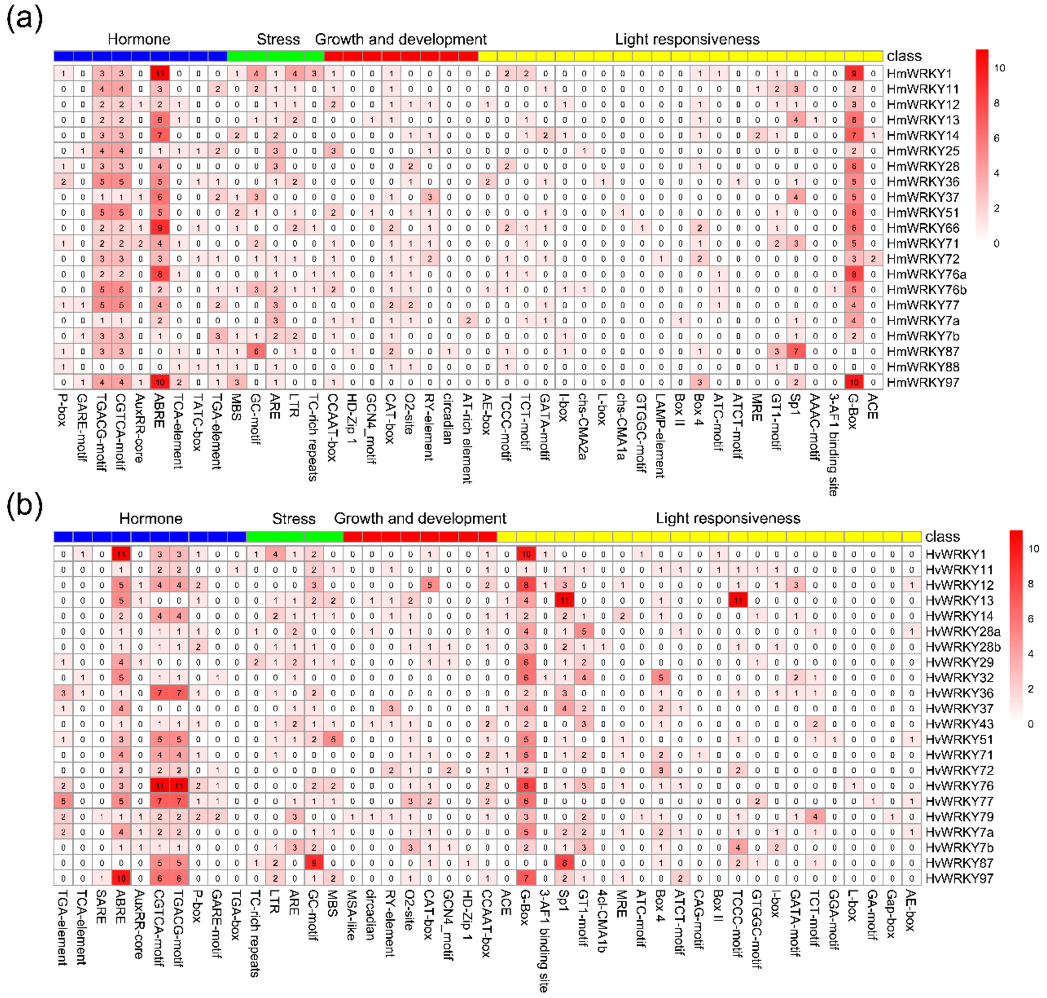

Figure S3. The distribution of cis-acting elements in the promoters of Group II *WRKY* genes in *H. vulgare* and *H. marinum*. Group II *WRKY* genes are shown on the right, and cis-acting elements—classified into four categories (hormone responses, stress responses, growth and development, and light responsiveness)—are listed at the bottom. The numbers within each block of the heatmap indicate the count of cis-acting elements present in the corresponding Group II *WRKY* gene, with a red color gradient representing element abundance.

(a)

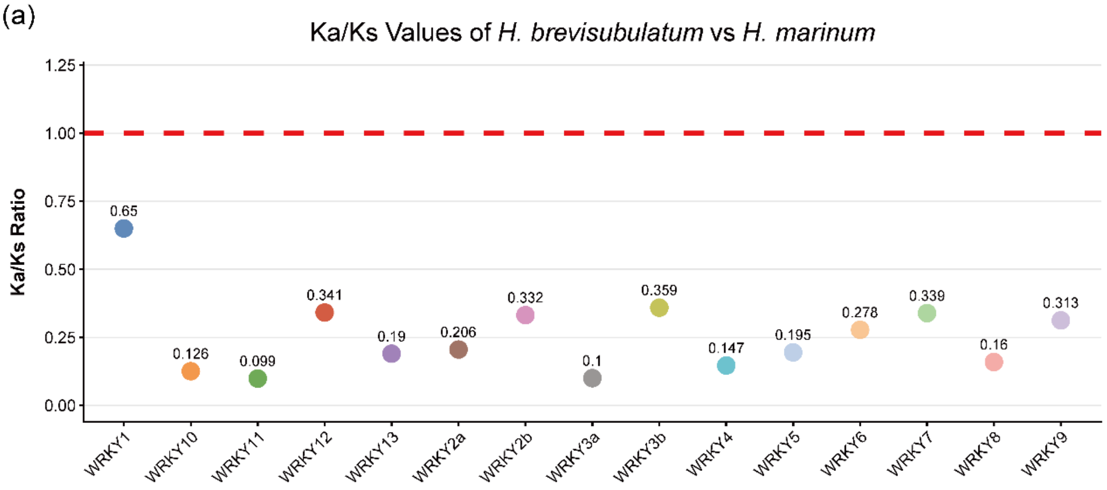

(b)

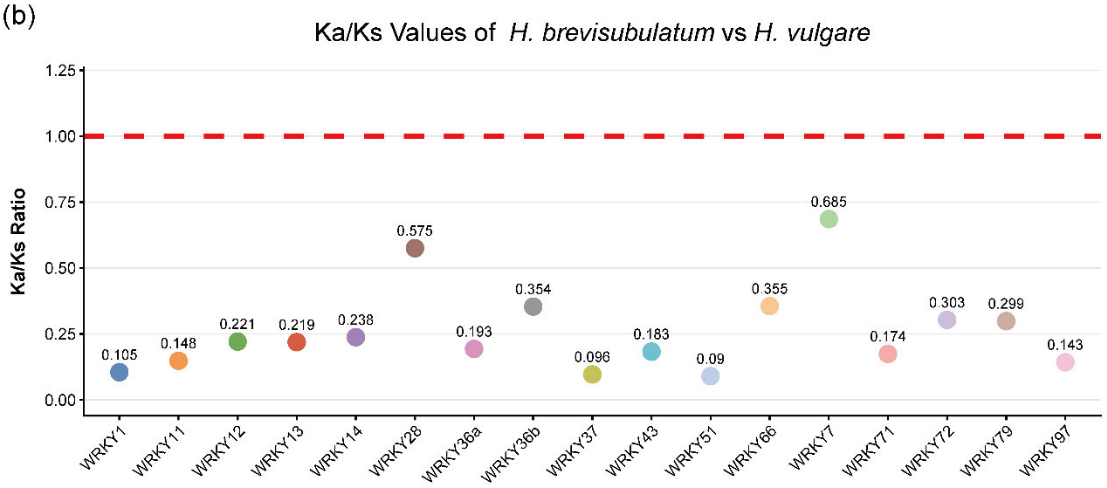

Figure S4. Ka/Ks ratios of orthologous Group II *WRKY* gene pairs between *H. brevisubulatum* and *H. marinum* (upper), and between *H. brevisubulatum* and *H. vulgare* (lower). The dashed red line indicates Ka/Ks = 1.

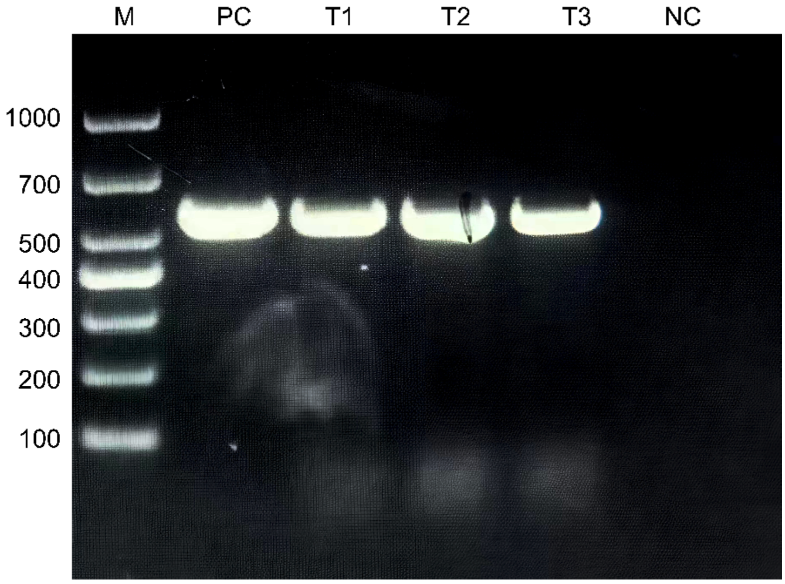

Figure S5. PCR identification of yeast transformants carrying *HbWRKY11*. Colony PCR was performed to verify the presence of the *HbWRKY11* insert in transformed yeast strains. Lane M: DNA marker 1000 bp; PC: positive control (pYES2-*HbWRKY11* plasmid); T1–T3: independent yeast transformants; NC: negative control (H_2O). A clear PCR band of the expected size (571 bp) was detected in PC and all transformants, while no amplification was observed in the negative control.
